# Increased mucosal CD4+ T-cell activation following vaccination with an adenoviral vector in rhesus macaques

**DOI:** 10.1186/1742-4690-9-S2-P267

**Published:** 2012-09-13

**Authors:** I Bukh, R Calcedo, S Roy, DG Carnathan, R Grant, SJ Ratcliffe, JM Wilson, MR Betts

**Affiliations:** 1 University of Pennsylvania, Philadelphia, PA, USA

## Background

The possibility that vaccination with Adenoviral vectors increased mucosal T-cell activation remains a central hypothesis to explain the potential enhancement of HIV acquisition within the STEP trial. Modeling this within rhesus macaques is complicated because human Adenoviruses, including Adenovirus type 5 (HAd5), do not productively infect macaques. We created a vector based upon a naturally occurring rhesus macaque Adenovirus (SAdV7) to test whether vaccination with a species-specific Adenoviral vector enhances mucosal T-cell activation within the natural host.

## Methods

Twelve rhesus macaques were vaccinated 3x intramuscularly with SAdV7 vector. Five HAd5-vaccinated animals were included as controls. PBMC and rectal biopsies were obtained at baseline, multiple times post-prime and post-17 week boost (8x/animal), and post-31 week second boost (1x/animal). We assessed rectal mucosal lamina propria and blood for frequency changes of Ad-specific T-cell responses and T-cell activation levels by measuring IFNg, TNFa, IL2, CD25, Ki67, CD69, and HLA-DR.

## Results

Naturally acquired pre-existing SAdV7-specific CD4+ T-cells were identified in 10/13 macaques within blood and/or rectal mucosa. Following intramuscular SAdV7 vaccination, rectal SAdV7-specific CD4+ T-cell responses increased above baseline in 9/9 animals 2-5 weeks post-prime, and subsequently contracted. Five weeks post-prime, 10/12 animals had rectal SAdV7-specific CD4+ T-cell responses ranging from 0.1-16.84%. As expected, SAdV7-specific CD4+ T-cells expressed CD69 and other activation markers (but not Ki67). Heightened expression of CD25, CD69, and HLA-DR was observed on total rectal memory CD4+ T-cells in SAdV7-vaccinated animals, and maintained 15 weeks after the prime. Interestingly, upregulation of activation markers in rectal mucosa also occurred in HAd5-vaccinated animals. No change in activation was observed in the blood throughout the entire study.

## Conclusion

These results indicate that peripheral vaccination with an Adenovirus vector can increase the activation of mucosal CD4+ T-cells providing an experimental model to further evaluate the role of host-vector interactions on increased HIV acquisition.

